# Peri-Myocarditis as a Dire Consequence of Coronavirus Disease: A Clinical Challenge

**DOI:** 10.7759/cureus.58739

**Published:** 2024-04-22

**Authors:** Ramya Pakala, Niyati Grewal, Jin Yi, Fatima Urooj

**Affiliations:** 1 Internal Medicine, Howard University Hospital, Washington, USA; 2 Cardiology, Howard University Hospital, Washington, USA

**Keywords:** coronavirus, cardiovascular disease, ventricular dysfunction, cardiomyopathy, myocarditis

## Abstract

Myocarditis is an inflammation of the heart muscle, most commonly caused by viral infections, with other contributing factors including medications or systemic inflammatory conditions. Coronavirus disease 2019 (COVID-19) is a disease caused by the severe acute respiratory syndrome coronavirus-2 (SARS-CoV-2 virus). In this report, we present a case of fulminant myocarditis in a patient with COVID-19 infection. Fulminant myocarditis is an aggressively progressive and severe variant that can result in substantial cardiac impairment. We present a case of fulminant myocarditis with a unique time course, progression, and potential challenges faced in diagnosis and management. Healthcare providers need to remain vigilant and anticipate the potential rapid progression of this disease.

## Introduction

Coronavirus disease 2019 (COVID-19)-associated myocarditis is a well-known manifestation of this disease. Cardiac involvement in COVID-19 infection is one of the early manifestations reported in the literature review, with presentations ranging from ventricular arrhythmia, acute coronary syndromes, myocardial involvement, and heart failure [1-5]. The incidence of myocarditis with COVID-19 infection has been increasing (from one to 10 cases per 100000 individuals to 150 to 4000 cases per 100000 individuals) [2]. The literature review reports a strong association between COVID-19 infection and peri-myocarditis [2-5]. Baskaran B et al. reported that 1.5% of patients with COVID-19 infection developed pericarditis and had a six-month all-cause mortality of 15.5% [6]. COVID-19 with peri-myocarditis can progress rapidly, becoming life-threatening. It is pertinent to identify the symptoms of acutely progressing heart failure and diagnostic markers of COVID-19 myocarditis: elevated troponins, inflammatory markers, pro-B-type natriuretic peptide (BNP), cardiac imaging, and biopsy to ensure treatment is initiated promptly [3-5]. Acute myocardial infarction should also be evaluated in these patients as it can present with similar clinical and diagnostic findings [3-5].

## Case presentation

A 60-year-old African American female with a medical history of hypertension, depression, and fibromyalgia arrived at the emergency department with complaints of sudden-onset chest pain and shortness of breath lasting for 36 hours. The patient experienced several episodes of chest pain, each episode lasted for 10 minutes, associated with episodes of chest tightness, shortness of breath, and diaphoresis. The patient did not report any fever, orthopnea, paroxysmal nocturnal dyspnea, palpitations, dizziness, falls, or loss of consciousness.

**Figure 1:**
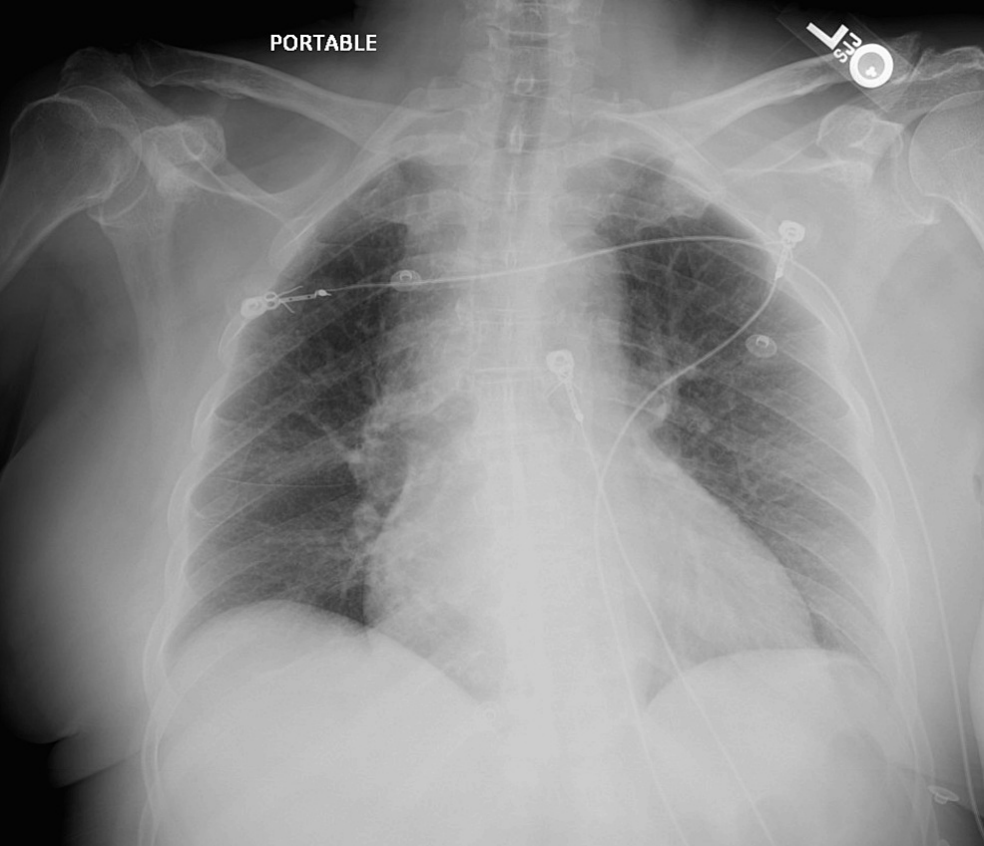
Chest X-ray (AP view) on the day of admission. Mild enlargement of the cardiac silhouette.

**Figure 2:**
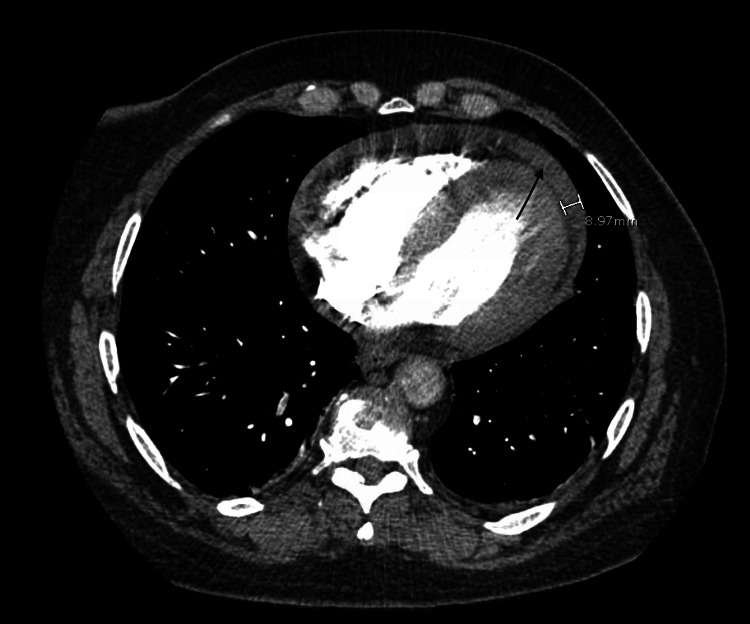
CT of the chest with IV contrast. The axial section shows a pericardial effusion marked by an arrow. CT: computed tomography; IV: intravenous

**Figure 3:**
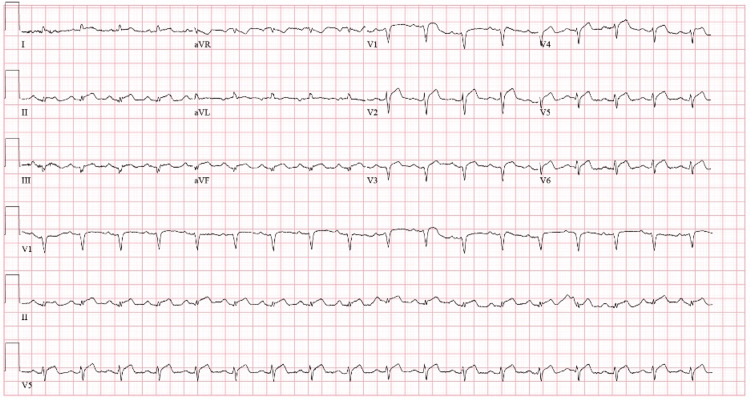
EKG on admission showing ventricular rate of 115 beats per minute, PR interval of 152 ms, QRS duration is 74 ms, QTC is 434 ms, diffuse ST elevation, sinus tachycardia, and low voltage QRS. EKG: electrocardiogram

On presentation, the patient had a temperature of 97.8°F, heart rate of 110 beats per minute, blood pressure of 125/88 mmHg, and oxygen saturation of 99% on room air. Initial blood investigations were significant for an elevated total leukocyte count (7.21x10^9^), elevated d-dimer (8.7 mg/mL), lactic acidosis of 10 mm/L, elevated troponin of 7.2 ng/mL, and repeat 10.1 ng/mL and elevated B-type natriuretic peptide (BNP) of 182 Pg/mL. She tested positive for COVID-19 on an antigen test. During the interview, the patient was vaccinated for COVID-19 with two doses of the initial series of vaccines. Chest X-ray revealed mild enlargement of the cardiac silhouette (Figure 1). Computed tomography (CT) of the chest with and without contrast was negative for pulmonary embolus, pertinent for pericardial effusion (9 mm in thickness), emphysematous changes, and atelectasis (Figure 2). An electrocardiogram (EKG) revealed sinus tachycardia with a ventricular rate of 115 beats per minute and low voltage QRS, along with diffuse ST elevations, indicating acute pericarditis (Figure 3). A bedside echocardiogram also showed pericardial effusion without tamponade. Due to initial suspicion of an acute coronary syndrome, the patient was administered clopidogrel (loading dose of 600 mg, followed by 75 mg once daily) and heparin infusion (60 units per kg loading dose, followed by infusion at 12 units/kg/hour titrated per aPTT). In addition, treatment for COVID-19 included remdesivir (200 mg IV one dose, followed by 100 mg IV daily for four days) and dexamethasone (6 mg orally once daily) therapy. A limited echocardiogram study revealed severe global hypokinesis of the left ventricle with an ejection fraction of less than 20%, the pericardial effusion was located anteriorly near the right atrium (RA), with moderate circumferential effusion.

On day one, a left and right heart catheterization procedure was performed that demonstrated angiographically patent coronaries, and left ventricular end-diastolic pressure (LVEDP) of 8 mmHg. The cardiac output by thermodilution was 2.26, two liters per minute by Fick’s, indicating a significant reduction in left ventricular function (Table 1 lists the hemodynamic measurements during the right heart catheterization procedure). This reduction in function with hypotension secondary to cardiomyopathy was diagnosed as non-ischemic and in the setting of a positive COVID-19 test, COVID-19-related myocarditis was high on the differential. The patient was initiated on goal-directed medical therapy for heart failure (sodium-glucose linked transporter two inhibitors (SGLT2i) and angiotensin receptor/neprilysin inhibitor). Serial troponins were obtained that were significant for uptrending levels of 13.76 ng/mL followed by 22.9 ng/mL.

**Table 1:** Hemodynamic measurements during right heart catheterization procedure. LVEDP: left ventricular end-diastolic pressure; PCWP: pulmonary venous wedge pressure; RA: Right atrium; RV: right ventricle; IV: intravenous; Ao: aortic; PA: pulmonary artery; TPG: transpulmonary pressure gradient; PVR: pulmonary vascular resistance

Parameters	Patient value	Reference range
Ao opening pressure	108/71 mmHg	SBP 100-140 mmHg, DBP 60-90 mmHg
LVEDP	8 mmHg	<12 mmHg
Post IV hydration PCWP	16 mmHg	8-12 mmHg
RA pressure	12 mmHg	1-5 mmHg
RV systolic pressure	29 mmHg	15-30 mmHg
RV diastolic pressure	10 mmHg	1-7 mmHg
PA systolic pressure	32 mmHg	15-30 mmHg
PA diastolic pressure	20 mmHg	4-12 mmHg
Mean PA pressure	26 mmHg	15 mmHg
TPG	10 mmHg	<=12 mmHg
Cardiac output by thermodilution	2.26 L/min	>5 L/min
Cardiac index	1.13 L/min/m^2^	>2.4 L/min/m^2^
PVR by thermodilution	4.4 WU	-0.17 WU

On day two, the patient developed significant hemodynamic instability with hypotension, that required pressor support. Dobutamine was initiated with the addition of norepinephrine to maintain a mean arterial pressure of more than 65. The patient was subsequently intubated for airway protection due to the poor Glasgow coma scale. On day three, she went into cardiac arrest with pulseless electrical activity with a return of spontaneous circulation after five minutes of advanced cardiac life support (ACLS). Soon after, she had another cardiac arrest with pulseless electrical activity, refractory to ACLS, and the patient passed. The autopsy was offered to the family; however, consent was denied. A viral panel for echovirus and coxsackievirus polymerase chain reaction (PCR) was sent for other noninfectious causes, which was negative, along with an atypical work-up for mycoplasma was negative. Blood cultures were negative. 

## Discussion

It has been reported that COVID-19 infection can cause severe cardiac manifestations, including myocarditis, arrhythmias, acute coronary syndrome, left ventricular heart failure, and acute and subacute pericarditis [1-5]. While the exact incidence of COVID-19-related myocarditis is unknown, studies suggest that approximately 7% of COVID-19-associated deaths are linked to this condition [1]. Additionally, a systematic review by Kariyanna T et al. found that COVID-19 pericarditis is more common in males, with a male-to-female ratio of two to one [4]. Myocarditis is typically caused by viral infections, such as adenovirus, enterovirus, and parvovirus [1].

Pericarditis is a condition where the pericardium becomes inflamed, either as a standalone issue or as a symptom of a larger systemic problem [2,4]. Myocardial injury caused by SARS-CoV virus occurs due to direct cell invasion, ischemic injury, and cytokine storm [2,4]. The pathophysiology of COVID-19-associated myocarditis involves angiotensin-converting enzyme-2 (ACE-2) receptors present in cardiac myocytes, which the virus uses to invade the cells. Once inside the cell, the virus impairs stress granule formation using its accessory protein. The virus then replicates and damages the cells in the absence of stress granules. Primed CD8+ T lymphocytes migrate to the cardiac myocytes and cause cell-mediated cytotoxicity, leading to phase one of viral myocarditis, which is associated with viral entry into the cell and replication. Phase two involves inflammatory cell infiltration, and phase 3 involves cardiac remodeling, which can lead to congestive heart failure secondary to myocarditis [1,2,7]. Myocarditis was also seen to develop after mRNA COVID-19 vaccination; however, the incidence was low, and it was reported within three to five days after vaccination [8]. Our patient was vaccinated with the COVID-19 vaccine a few months prior to this presentation (unsure of vaccine type and exact timing since the last vaccine).

Research indicates that ischemic injury can occur due to respiratory infections causing an imbalance between oxygen supply and demand, ultimately leading to decreased myocardial oxygen supply [2,4]. Additionally, the virus can trigger a cytokine storm, which is a widespread cytokine release throughout the body that can contribute to myocardial damage [2,4]. Current literature suggests that COVID-19 has not been isolated from cardiac myocytes, indicating the virus does not infect cardiac myocytes directly; instead, it triggers an inflammatory immune response, leading to myocarditis and inflammation in the adjacent pericardium [1]. This inflammation can result in pericarditis and effusion occurring simultaneously, potentially leading to tamponade if the effusion is abrupt and reduces filling and cardiac output [1]. COVID-19-induced pericarditis commonly manifests as chest pain, fever, shortness of breath, cough, fatigue, myalgias, and diarrhea [4,5]. Physicians identify cardiac tamponade by examining Beck's triad (hypotension, jugular venous distension, and muffled heart sounds) and pulsus paradoxes [1,3].

Blood tests conducted on individuals with COVID-19 pericarditis often show an increase in white blood cells with a higher count of neutrophils, elevated levels of d-dimer, erythrocyte sedimentation rate, and C-reactive protein, as well as heightened levels of troponin and BNP [4,5]. Diagnosis of pericarditis may involve EKG results showing diffuse ST elevation, PR depression, and low voltage QRS complexes, along with an EKG revealing a collapsed RA and ventricle during diastole [1,3]. For COVID-19 patients experiencing hemodynamic instability or cardiac symptoms, an early focused cardiac ultrasound may aid in identifying cardiac tamponade [9-11].

To diagnose COVID-19 pericarditis, it is recommended to utilize CT and cardiac magnetic resonance imaging (MRI) with gadolinium to detect pericardial inflammation and assess pericardial thickening. It is important to note that although cardiac MRI may be limited, it remains a valuable diagnostic tool [3-5]. Chest X-rays should not be solely relied upon as 31% of cases showed cardiomegaly with or without bilateral infiltration [3-5]. CT scans are also useful in identifying evidence of myocarditis through an increase in wall thickness, myocardial edema, and wall hypokinesis [3-5]. Over the years non-invasive modalities of the diagnosis have taken precedence over invasive modalities like endomyocardial biopsy, which are reserved in fulminant cases or in patients not responding to empiric treatment [5]. The diagnostic yield of biopsy can be improved by using electroanatomic mapping (EAM) or fluoroscopy [11].

According to a review of the literature on COVID-19 pericarditis cases, the preferred treatment approach involves the use of colchicine, followed by non-steroidal anti-inflammatory drugs (NSAIDs) and aspirin [3-5]. However, in myocarditis-related heart failure and cardiogenic shock, NSAIDs can cause sodium retention and worsen volume overload [3]. For myocarditis-related heart failure, early initiation of management aimed at treating cardiogenic should be initiated: inotropes, vasopressors, and mechanical ventilation [3]. In cases where patients are unresponsive to or unable to tolerate aspirin, steroids may be used [3,4]. However, there is no evidence to support the use of intensive immunosuppression or direct antiviral therapy in peri-myocarditis due to COVID-19 [3,9]. If there is a high clinical suspicion of tamponade, it is important to consider emergent pericardiocentesis or pericardial window as they can improve mortality [1,9]. Tamponade can lead to a rapid decline in the hemodynamic status of these patients and, as such, the diagnosis should have a low threshold and emergent treatment should be prioritized [1]. If cytokine storm is suspected, monoclonal antibodies, namely tocilizumab or sarilumab (interleukin-16 antagonist) should be considered [3]. 

Data indicates COVID-19 pericarditis patients have mostly recovered or not experienced further clinical decline [3-5]. However, certain factors such as fever, subacute course, large effusion or tamponade, elevated troponin, and natriuretic peptides can indicate a poor prognosis [4-9]. It is important to note that peri-myocarditis patients are at a higher risk of complications, including left ventricular dysfunction and heart failure [4]. The literature reported only a few deaths, all of which were related to peri-myocarditis [4]. Long-term complications of recovered myocardial involvement are unknown and further studies are needed [3].

## Conclusions

Fulminant myocarditis refers to a rapidly progressive and severe form of myocarditis that can lead to significant cardiac dysfunction and potentially be life-threatening. It can occur as a complication of COVID-19 infection and may progress quickly, as was the case in our situation. Prompt and aggressive treatment is essential in cases of fulminant myocarditis. The primary goal is to stabilize the patient's condition, reduce inflammation, and support cardiac function.

This case underscores the need for further research to enhance our understanding of this condition, improve diagnostic approaches, refine treatment strategies, and optimize patient outcomes. Healthcare providers can enhance the management of fulminant myocarditis linked with COVID-19 by exchanging experiences and studying comparable cases.
